# Rethinking aerobic exercise intensity prescription in adults with spinal cord injury: time to end the use of “moderate to vigorous” intensity?

**DOI:** 10.1038/s41393-021-00733-2

**Published:** 2021-12-08

**Authors:** Michael J. Hutchinson, Victoria L. Goosey-Tolfrey

**Affiliations:** Peter Harrison Centre for Disability Sport, School of Sport, Exercise and Health Sciences, Loughborough University, Loughborough, UK

**Keywords:** Physiology, Health occupations

## Abstract

**Study design:**

Cohort study.

**Objectives:**

To investigate and critique different methods for aerobic exercise intensity prescription in adults with spinal cord injury (SCI).

**Setting:**

University laboratory in Loughborough, UK.

**Methods:**

Trained athletes were split into those with paraplegia (PARA; *n* = 47), tetraplegia (TETRA; *n* = 20) or alternate health condition (NON-SCI; *n* = 67). Participants completed a submaximal step test with 3 min stages, followed by graded exercise test to exhaustion. Handcycling, arm crank ergometry or wheelchair propulsion were performed depending on the sport of the participant. Oxygen uptake (V̇O_2_), heart rate (HR), blood lactate concentration ([BLa]) and ratings of perceived exertion (RPE) on Borg’s RPE scale were measured throughout. Lactate thresholds were identified according to log-V̇O_2_ plotted against log-[BLa] (LT_1_) and 1.5 mmol L^−1^ greater than LT_1_ (LT_2_). These were used to demarcate moderate (<LT_1_), heavy (>LT_1_, < LT_2_) and severe (>LT_2_) exercise intensity domains.

**Results:**

Associations between percentage of peak V̇O_2_ (%V̇O_2peak_) and HR (%HR_peak_) with RPE differed between PARA and TETRA. At LT_1_ and LT_2_, %V̇O_2peak_ and %HR_peak_ were significantly greater in TETRA compared to PARA and NON-SCI (*P* < 0.05). The variation in %V̇O_2peak_ and %HR_peak_ at lactate thresholds resulted in large variability in the domain distribution at fixed %V̇O_2peak_ and %HR_peak_.

**Conclusions:**

Fixed %V̇O_2peak_ and %HR_peak_ should not be used for aerobic exercise intensity prescription in adults with SCI as the method does not lead to uniform exercise intensity domain distribution.

## Introduction

For adults with spinal cord injury (SCI), aerobic exercise is beneficial for improving indices of physical [1] and mental [2] health. On this theme, scientific guidelines published in 2018 describe the dose of aerobic exercise required to improve cardiorespiratory fitness and cardiometabolic health in adults with SCI [3]. Central to the guidelines is information on the frequency (e.g., 3 times per week) and duration (e.g., 30 min) of the exercise, both of which are simple to define and monitor. The final important aspect of the guidelines is the exercise intensity. If aerobic exercise is performed at too low an intensity, without sufficient exercise volume, it will not lead to beneficial physiological adaptations [4]. Despite this, the guidelines provide no clear prescription of the exercise intensity, other than to say that aerobic exercise should be of a “moderate to vigorous” intensity [3]. The lack of clarity with the exercise intensity terminology is a hindrance to adults with SCI using the guidelines to inform their exercise habits; practitioners actively prescribing exercise training; and researchers investigating the effects of exercise training interventions on markers of health in adults with SCI. There is, therefore, an urgent need to better understand aerobic exercise intensity prescription in adults with SCI.

Guidelines for non-disabled adults define thresholds for five intensity zones (very light, light, moderate, vigorous, near-maximal/maximal) according to many physiological variables [5]. These variables include percentage maximum oxygen uptake (%V̇O_2max_) and heart rate (%HR_max_), oxygen uptake and heart rate reserve (%V̇O_2_R, %HRR), and ratings of perceived exertion (RPE) [5]. However, despite the SCI guidelines adopting the “moderate” and “vigorous” descriptives, there is no equivalent resource published for adults with SCI regarding the physiological thresholds coinciding with these descriptors. Furthermore, given the physiological consequences of SCI on cardiovascular and respiratory responses to exercise [6], there is no justification for simply adopting the percentage thresholds utilised for non-disabled adults.

An alternative approach to exercise intensity prescription is to consider whether different methods result in participants exercising in the same of three exercise domains (moderate, heavy, severe) [7]. This is because of the similar V̇O_2_ and blood lactate responses between individuals exercising in these domains [7]. Specifically, the moderate intensity domain (below lactate threshold (LT)) is characterised by steady state responses for V̇O_2_ and blood lactate concentration ([BLa]) [8]. In the heavy intensity domain (between LT and critical power/speed (CP/CS)) there is a delayed steady state response due to the V̇O_2_ “slow component”, whilst in the severe domain (above CP/CS) no steady state response is observed [8].

To satisfy the aim of producing a homogenous exercise intensity, the fixed percentage approach is only valid if it is demonstrated that equal relative intensities result in individuals exercising in the same intensity domain [7]. However, in a recent study of non-disabled participants, no fixed %V̇O_2max_ or %HR_max_, typically used for exercise prescription, resulted in all participants being in the same intensity domain [9]. This has led to assertions that using fixed %V̇O_2max_ or %HR_max_ for prescribing exercise intensity is inaccurate and will lead to significant inter-individual physiological responses, precluding homogenous exercise intensity prescription [7, 9]. Furthermore, with evidence that individual participant %V̇O_2_R:%HRR relationships diverges from the assumed linear trajectory, there are also questions over how appropriate %V̇O_2_R and %HRR are for prescribing exercise intensity at the individual level [10].

For adults with SCI there is currently nothing more to inform aerobic exercise intensity prescription than the arbitrary use of “moderate to vigorous” intensity [3]. Furthermore, evidence in non-disabled adults would suggest a need to rethink the traditional use of fixed percentages [7, 9, 10]. Therefore, this study aimed to investigate and critique potential methods for prescribing aerobic exercise intensity in adults with SCI.

## Methods

This study was performed via a retrospective analysis of athlete data collected in the author’s laboratory. All procedures were approved by the Human participants ethical sub-committee at Loughborough University, and participants provided written, informed consent.

### Participants

Data were available for 134 individuals (male: 98; female: 36). Participants were split into those with paraplegia (PARA), tetraplegia (TETRA), or alternate health condition (NON-SCI), see Table 1. Examples of health conditions for NON-SCI included spina bifida, limb deficiency, cerebral palsy, and arthrogryposis. Participants were competitive athletes, competing at a national or international level, from one of the following sports: handcycling, para-alpine ski, paratriathlon, wheelchair basketball, wheelchair rugby or wheelchair tennis.

**Table 1:** Participant characteristics by group.

		PARA	TETRA	NON-SCI
Sample size (n)		47	20	67
Sex (M/F)		30/17	18/2	50/17
Age (years)		33 ± 8^a^	32 ± 7^a^	27 ± 7
Body mass (kg)		70.9 ± 14.1^a^	70.8 ± 12.9	64.5 ± 12.8
Neurological level of injury		T4-L2	C3-C7	-
Injury completeness		Complete: 21	Complete: 8	-
		Incomplete: 23	Incomplete: 4	
		Unavailable: 3	Unavailable: 8	
Time since injury (years)		12 ± 9	12 ± 6	-
Peak oxygen uptake	(L·min^−1^)	2.5 ± 0.6^b^	1.7 ± 0.5	2.6 ± 0.7^b^
	(ml·kg^−1^·min^−1^)	35.1 ± 8.2^b^	23.4 ± 5.8	39.9 ± 8.3^b^
Peak heart rate	(beats·min^**−1**^)	188 ± 9^b^	134 ± 20	187 ± 10 ^b^
Sport (n)	Handcycling	8	0	3
	Paratriathlon	11	0	8
	Para alpine ski	2	1	2
	Wheelchair basketball	20	1	31
	Wheelchair rugby	0	15	13
	Wheelchair tennis	6	3	10
Test mode (n)	Arm crank ergometry	11	1	7
	Handcycling	10	0	6
	Wheelchair propulsion	26	19	54

^a^: significantly greater than NON-SCI; ^b^: significantly greater than TETRA, *P* < 0.05.

### Exercise testing

Participants completed a submaximal step test followed by graded exercise test (GXT) to exhaustion. Handcycle (HC) tests were performed in the participants own handcycle attached to a Cyclus 2 ergometer (Avantronic Richter, Leipzig, Germany). For some Paratriathlon, and all para-alpine ski athletes, arm crank ergometry (ACE) was used (Lode Angio, Lode B. V., Groningen, the Netherlands). The ergometer was positioned vertically so the crank axis centre was level with the shoulder, and horizontally to allow slight elbow flexion at the furthest point of the crank cycle. Wheelchair basketball, rugby and tennis players performed a wheelchair propulsion (WCP) test using a motorised treadmill (HP Cosmos, Traunstein, Germany) and their own custom sports wheelchair.

Submaximal tests were individualised based on the sport, sex, training status and level of impairment of the participant, with the goal of completing 6-8 stages (average: 6; range: 4–10). HC and ACE tests started at 15–60 W, with 10–20 W increments every 3 min. WCP tests started at 0.7–2.8 m s^−1^ and were increased by 0.2-0–4 m s^−1^ every 3 min. V̇O_2_ (Metalyzer 3B, Cortex, Leipzig, Germany) and HR (RS400, Polar, Kempele, Finland) were continually monitored throughout. The Metalyzer was calibrated before each participant against ambient air and a mix of 15% O_2_, 5% CO_2_, with the volume calibrated using a 3 L syringe. RPE was verbally reported in the final minute of each stage using Borg’s 6–20 RPE scale [11]. A capillary blood sample from the ear lobe was collected at the end of each stage for measurement of [BLa] (Biosen C-line, EKF Diagnostics, Barleben, Germany). HC and ACE tests were continuous, however, WCP tests were discontinuous as the treadmill needed to be slowed between stages to facilitate blood sampling. For discontinuous tests, the typical interval between stages was 45–60 s. Submaximal tests continued until [BLa] exceeded 4 mmol L^−1^ or RPE was rated as 17. The RPE criteria was used in TETRA where there may have been blunted lactate responses [12].

Following the submaximal test, participants received 15 min of active recovery or rest before performing a GXT to exhaustion. The starting workload was set to that from the preceding test when [BLa] increased by 0.5 mmol L^−1^ above rest. Participants performed 1 min at this load, before the exercise intensity were increased in a stepwise manner by 10–20 W min^−1^ (HC/ACE) or 0.1 m s^−1^ min^−1^ (WCP) until participants reached volitional exhaustion. This was defined as an inability to maintain their preferred cadence at the required PO for HC/ACE, or the required speed of the treadmill, despite verbal encouragement. V̇O_2_ and HR were again monitored throughout, with RPE and [BLa] measured at the end of the test.

### Data processing

V̇O_2_ and HR data were subjected to a 30 s rolling average, with the greatest of these from the GXT recorded as peak values (V̇O_2peak_, HR_peak_). V̇O_2_ and HR in the final 30 s of each submaximal stage were extracted and calculated as percentages of peak (%V̇O_2peak_, %HR_peak_). Using the submaximal data, the lactate thresholds were identified as the intersection of the horizontal and ascending sections of the plot of log-[BLa] against log-V̇O_2_ (LT_1_) [13], and at [BLa] equal to LT_1_ plus 1.5 mmol L^−1^ (LT_2_) [14]. The inverse of the log-V̇O_2_ at these points were calculated to give the V̇O_2_ at LT_1_ and LT_2_. HR at LT_1_ and LT_2_ was identified by interpolation of the linear V̇O_2_:HR relationship for each participant. RPE was modelled against [BLa] using a quadratic function for each participant, with the resultant coefficients used to calculate the RPE at LT_1_ and LT_2_ [15]. Exercise intensity domains were defined as moderate (<LT_1_), heavy (between LT_1_ and LT_2_) and severe (>LT_2_).

### Statistical analyses

Analyses were performed using IBM SPSS Statistics Version 23.0 (IBM Corp., Armonk, NY) and MLWiN Version 3.05 [16]. Data are presented as mean (standard deviation) with statistical significance accepted at *P* < 0.05. Data were checked for normal distribution using the Shapiro Wilk statistic.

All individual RPE data points were modelled against the corresponding %V̇O_2peak_ and %HR_peak_ using dynamic multilevel models with lagged independent variable, whilst accounting for the initial condition. Separate models were created for %V̇O_2peak_ and %HR_peak_, which served as the independent variable, with RPE as the dependent variable. Models were multilevel to adjust for the repeated stages performed by each participant and were used due to their ability to characterise group- and individual-level effects [17]. Stage was defined as the first, and participant as the second level. Models accounted for the initial condition (e.g., stage (i) = 1) as it was thought that RPE would depend on the %V̇O_2peak_ and %HR_peak_ when i = 1. The need for them to be dynamic and incorporate a lagged independent variable was required as it was thought RPE at subsequent measurement occasions (when i > 1) would be dependent on %V̇O_2peak_ and %HR_peak_ for that, as well as the previous, measurement occasion (i.e., i–1). Potential confounding variables were added to the models to assess whether they improved the model fit with fixed effects, or random effects for between- and within-individual variation. Confounding variables were sex (male/female), group (PARA/TETRA/NON-SCI) and exercise mode (ACE/HC/WCP). The resultant models were used to calculate the %V̇O_2peak_ and %HR_peak_ corresponding to each value on Borg’s RPE scale.

Differences in V̇O_2_ (L min^−1^, ml kg^−1^ min^−1^, %V̇O_2peak_), HR (beats·min^−1^, %HR_peak_) and RPE between groups at LT_1_ and LT_2_ were assessed via one-way analysis of variance with Bonferroni post-hoc correction for multiple comparisons. Standardised effect sizes (ES) were calculated to describe the magnitude of differences and categorised as trivial (< 0.2), small (0.2–0.6), moderate (0.6–1.2), large (1.2–2.0) and very large (> 2.0) [18]. For each group, the percentage of participants in each intensity domain (moderate, heavy, severe) were calculated at 5% intervals from 35 to 95% V̇O_2peak_ and %HR_peak_.

## Results

The associations between RPE and both %V̇O_2peak_ and %HR_peak_ were not significantly affected by sex or exercise mode, so stratification based on these variables was not needed. The %V̇O_2peak_ and %HR_peak_ coinciding with each rating on Borg’s RPE scale for PARA and TETRA can be found in Table 2. The full RPE models against %V̇O_2peak_ and %HR_peak_ can be found in the Supplementary Material.

**Table 2:** Resultant calculations of percentage peak oxygen uptake and heart rate by group based on multilevel modelling.

RPE	%V̇O_2peak_		%HR_peak_	
	PARA	TETRA	PARA	TETRA
6	16	22	40	43
7	22	28	44	48
8	28	34	49	52
9	34	40	54	57
10	40	46	58	62
11	46	52	63	66
12	52	58	68	71
13	58	64	73	76
14	64	70	77	81
15	70	76	82	85
16	76	82	87	90
17	82	88	91	95
18	88	94	96	99
19	94	100	100	
20	100			

### RPE and %V̇O_2peak_ model

RPE was significantly affected by the initial %V̇O_2peak_ (i = 1) (*P* < 0.01), by %V̇O_2peak_ at subsequent occasions when i > 1 (*P* < 0.01), as well as by the lagged %V̇O_2peak_ (i.e., i–1) (*P* < 0.01). Each of these variables also showed significant between-individual variation, which was incorporated into the model. There was also an effect of Group at occasions when i > 1 (*P* = 0.01). TETRA showing significantly greater within-individual variation for the effect of %V̇O_2peak_ on RPE compared to PARA and NON-SCI. As such, PARA and NON-SCI remained grouped, as there was no difference between these groups.

### RPE and %HR_peak_ model

RPE was significantly affected by the initial %HR_peak_ (i = 1) (*P* < 0.01), by %HR_peak_ at subsequent occasions when i > 1 (*P* < 0.01), as well as by the lagged %HR_peak_ (i.e., i–1) (*P* < 0.01). These effects were fixed and showed no significant between- or within-individual variation. There was a fixed effect for Group, with the association between RPE and %HR_peak_ being significantly different for PARA (*P* = 0.03). There was no difference between TETRA and NON-SCI, so these remained grouped in this model.

### Responses at LT_1_ and LT_2_

**Fig. 1:**
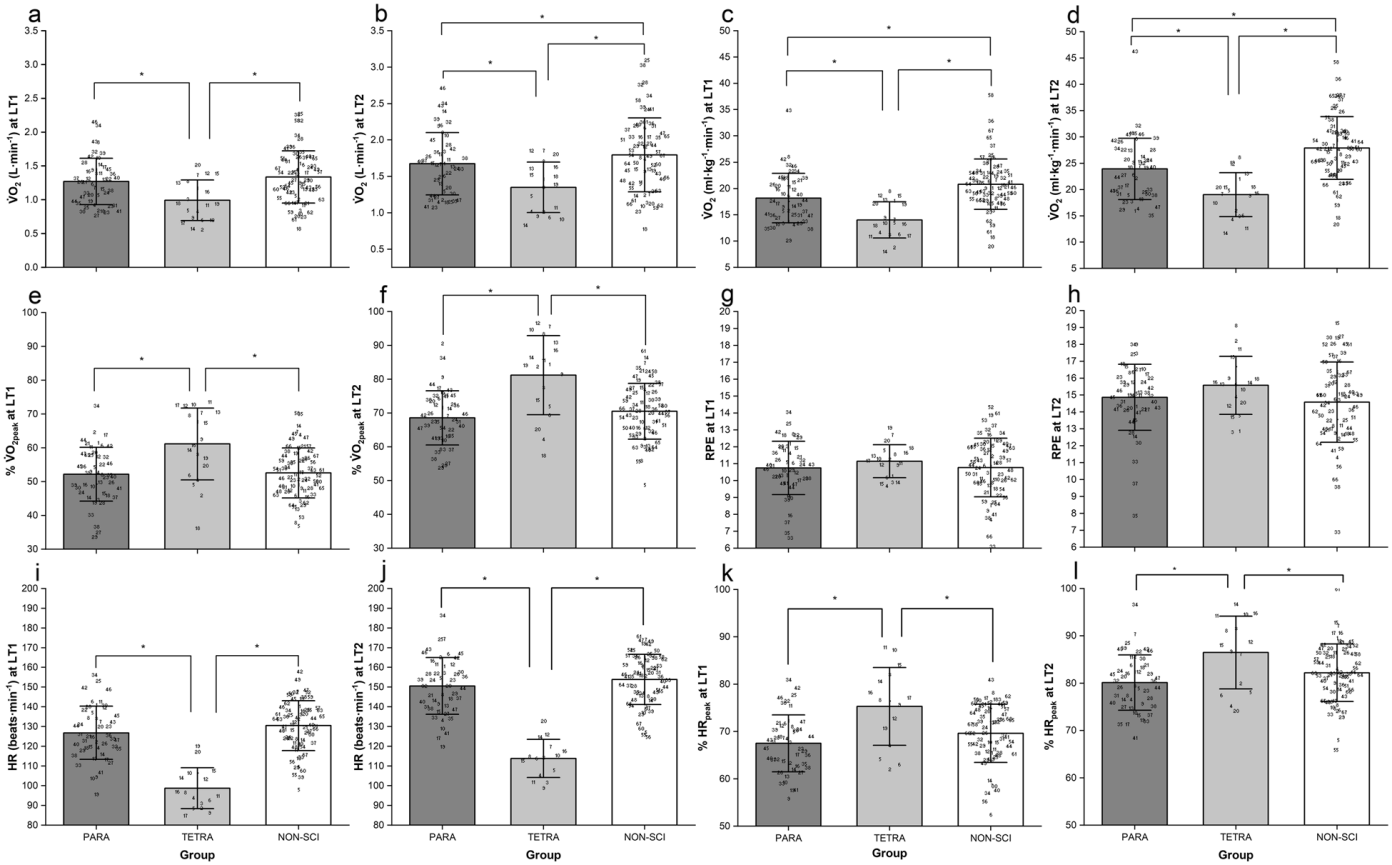
Group physiological responses at lactate thresholds. **a** Absolute V̇O2 at LT1. **b** Absolute V̇O2 at LT2. **c** Relative V̇O2 at LT1. **d** Relative V̇O2 at LT2. **e** %V̇O2peak at LT1. **f** %V̇O2peak at LT2. **g** RPE at LT1. **h** RPE at LT2. **i** HR at LT1. **j** HR at LT2. **k** %HRpeak at LT1. **l** %HRpeak at LT2. Data are presented at mean (SD) with individual points overlaid. Within each group, numbers refer to the same participant in each figure. Asterisk (*) indicates significantly greater than the identified group, *P* < 0.05.

The V̇O_2_, HR and RPE at LT_1_ and LT_2_ are shown in Fig. 1. At LT_1_ there was a significant group effect for absolute (F_2_ = 7.11, *P* < 0.01; Fig. 1a) and relative V̇O_2_ (F_2_ = 17.65, *P* < 0.01; Fig. 1c), %V̇O_2peak_ (F_2_ = 9.86, *P* < 0.01; Fig. 1e), HR (F_2_ = 42.79, *P* < 0.01; Fig. 1i) and %HR_peak_ (F_2_ = 7.94, *P* < 0.01; Fig. 1k). LT_1_ occurred at a significantly smaller absolute and relative V̇O_2_ in TETRA compared to PARA (ES = 0.86, 1.00) and NON-SCI (ES = 1.00, 1.62). However, %V̇O_2peak_ at LT_1_ was significantly greater in TETRA compared to PARA (ES = 0.96) and NON-SCI (ES = 0.94). Similarly, HR at LT_1_ was smaller in TETRA compared to PARA (ES = 2.33) and NON-SCI (ES = 2.74), whereas %HR_peak_ was greater in TETRA compared to PARA (ES = 1.00) and NON-SCI (ES = 0.69). There was no significant difference between groups for RPE (F_2_ = 0.48, *P* = 0.62) at LT_1_ (Fig. 1g).

There was also a significant group effect at LT_2_ for absolute (F_2_ = 9.96, *P* < 0.01; Fig. 1b) and relative V̇O_2_ (F_2_ = 19.75, *P* < 0.01; Fig. 1d), %V̇O_2peak_ (F_2_ = 14.80, *P* < 0.01; Fig. 1f), HR (F_2_ = 58.99, *P* < 0.01; Fig. 1i) and %HR_peak_ (F_2_ = 6.10, *P* < 0.01; Fig. 1l). Absolute and relative V̇O_2_ at LT_2_ were significantly smaller in TETRA compared to PARA (ES = 0.83, 0.97) and NON-SCI (ES = 1.03, 1.72). However, %V̇O_2peak_ at LT_2_ was significantly greater in TETRA compared to PARA (ES = 1.26) and NON-SCI (ES = 1.06). Furthermore, HR at LT_2_ was significantly smaller in TETRA than in PARA (ES = 3.00) and NON-SCI (ES = 3.55), while %HR_peak_ was significantly greater in TETRA compared to PARA (ES = 0.93) and NON-SCI (ES = 0.62). There was no significant difference between groups in RPE (F_2_ = 2.18, *P* = 0.19) at LT_2_ (Fig. 1h).

### Intensity classification

**Fig. 2:**
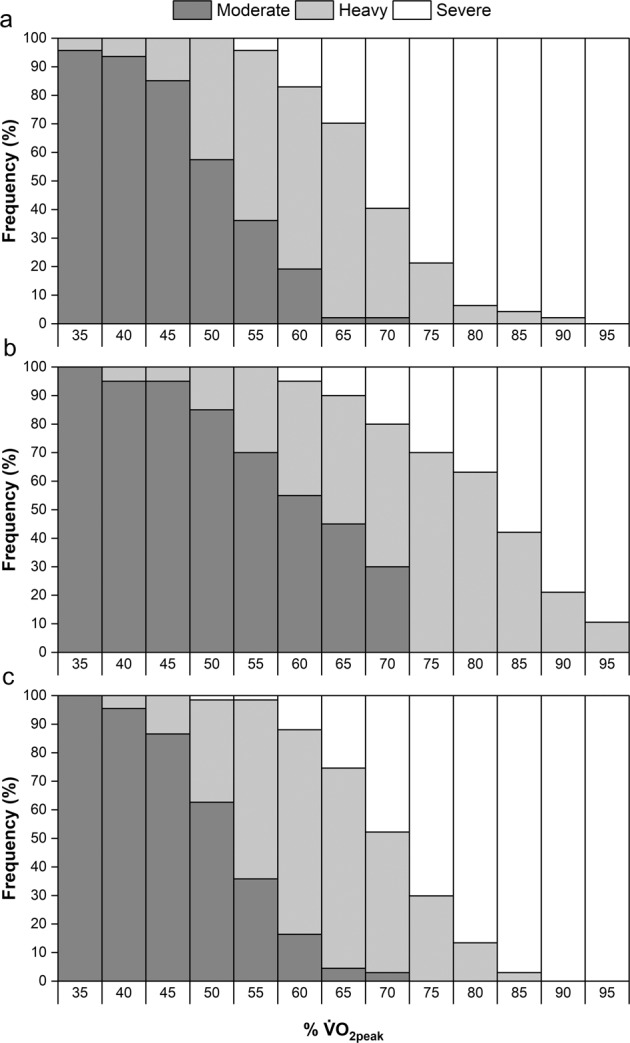
Distribution of individuals in moderate, heavy, and severe intensity domains at fixed %V̇O2peak. **a** PARA group. **b** TETRA group. **c** NON-SCI group.

**Fig. 3:**
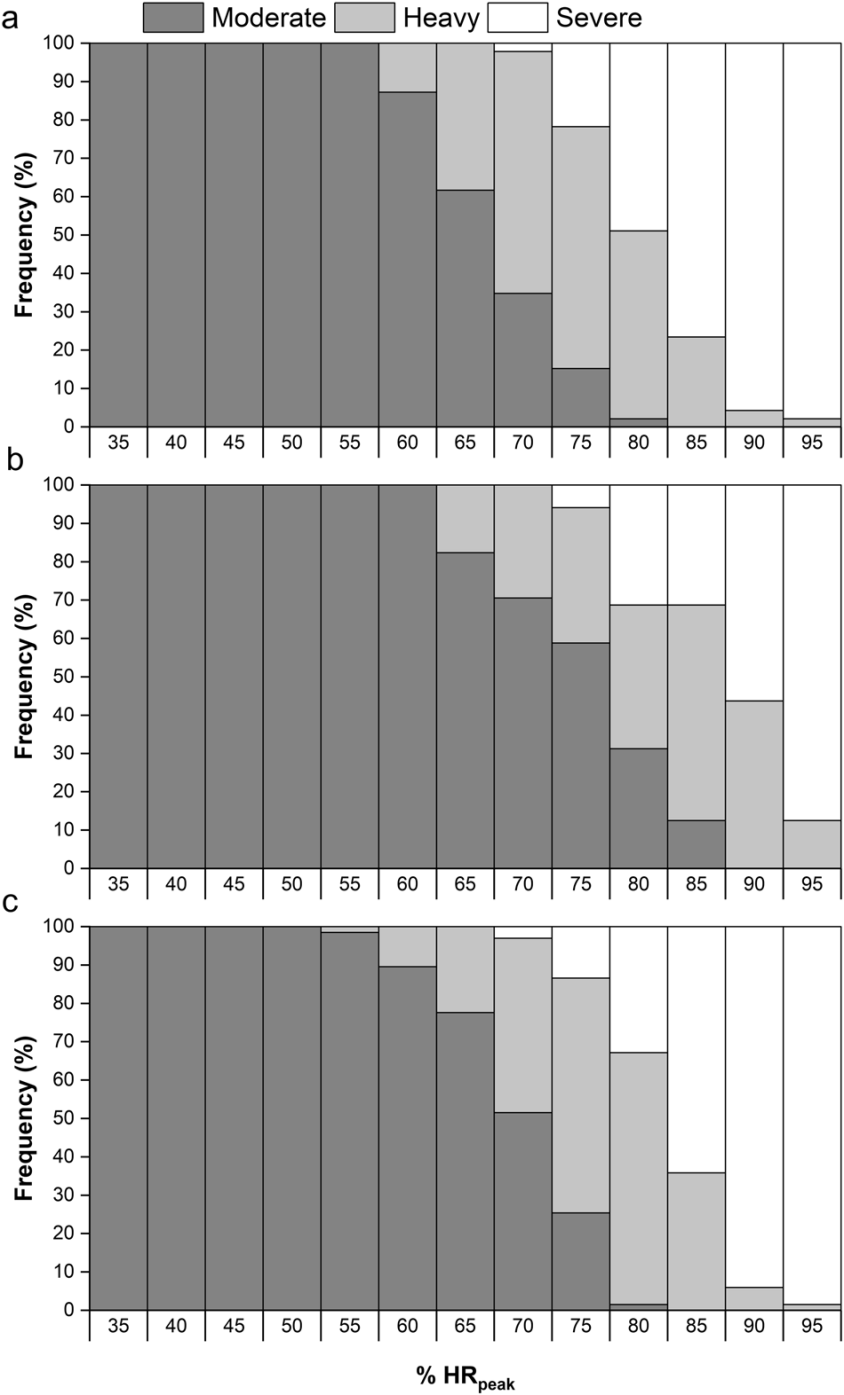
Distribution of individuals in moderate, heavy and severe intensity domains at fixed %HRpeak. **a** PARA group. **b** TETRA group. **c** NON-SCI group.

Thresholds for %V̇O_2peak_ and %HR_peak_ corresponding with intensity classifications used in non-disabled exercise guidelines are shown in Table 3. These data suggest there are differences between non-disabled individuals, PARA and TETRA in the thresholds for intensity classifications. Frequency distribution of individuals within moderate, heavy and severe intensity domains for discrete percentages of %V̇O_2peak_ and %HR_peak_ are shown in Figs. 2 and 3, respectively. These show that no %V̇O_2peak_ or %HR_peak_ typically used for exercise prescription purposes leads to all participants being in the same domain, with many %V̇O_2peak_ including participants spread across all three domains.

**Table 3:** Classification of exercise intensity for individuals with paraplegia and tetraplegia, compared to non-disabled guidelines.

	Very light (RPE ≤ 8)^a^	Light (RPE 9–11)^a^	Moderate (RPE 12–13)^a^	Vigorous (RPE 14–17)^a^	Near maximal-maximal (RPE ≥ 18)^a^
%V̇O_2peak_					
Non-disabled^a^	≤36% V̇O_2peak_	37–45% V̇O_2peak_	46–63% V̇O_2peak_	64–90% V̇O_2peak_	≥91% V̇O_2peak_
Paraplegia	≤31% V̇O_2peak_	32–49% V̇O_2peak_	50–61% V̇O_2peak_	62–85% V̇O_2peak_	≥86% V̇O_2peak_
Tetraplegia	≤37% V̇O_2peak_	38-55% V̇O_2peak_	56–67% V̇O_2peak_	68–91% V̇O_2peak_	≥92% V̇O_2peak_
%HR_peak_					
Non-disabled^a^	≤56% HR_peak_	57–63% HR_peak_	64-76% HR_peak_	77–95% HR_peak_	≥96% HR_peak_
Paraplegia	≤51% HR_peak_	52–65% HR_peak_	66-75% HR_peak_	76–93% HR_peak_	≥94% HR_peak_
Tetraplegia	≤54% HR_peak_	55–68% HR_peak_	69–78% HR_peak_	79–97% HR_peak_	≥98% HR_peak_

^a^ data from Riebe et al. [5].

## Discussion

This study aimed to investigate potential methods of aerobic exercise intensity prescription in adults with SCI. Findings demonstrate that there are differences between PARA and TETRA for the %V̇O_2peak_ and %HR_peak_ corresponding with the descriptions of “moderate” and “vigorous” exercise intensity, as used by the exercise guidelines for adults with SCI [1, 3]. However, the findings also show that using fixed %V̇O_2peak_ or %HR_peak_ cannot guarantee a homogenous domain-specific exercise intensity prescription for adults with SCI.

### Fixed percentages and intensity domains for exercise intensity prescription

The finding of adults with SCI being in different intensity domains, as defined in this study according to LT_1_ and LT_2_, despite being at the same %V̇O_2peak_ or %HR_peak_ supports similar evidence in non-disabled participants [9]. The domain-specific distribution is also arguably more variable in adults with SCI. For V̇O_2peak_, Iannetta et al. [9]. report participants in moderate, heavy, and severe domains only at 70% V̇O_2peak_, whereas in this study this was shown at several fixed percentages, 55-70% V̇O_2peak_ in PARA and 60-70% V̇O_2peak_ in TETRA. It would, therefore, given recent calls to stop using fixed percentages for exercise intensity prescription in non-disabled participants [7, 9, 10], seem appropriate that this is expanded to apply to adults with SCI performing aerobic exercise. This would apply to all adults with SCI, and not just the athletic population utilised in this study. Inter-individual variation will exist in sedentary or low-active participants as much, if not more, than in athletic groups, further limiting the use of fixed percentages for exercise intensity prescription.

Instead, more attention should be given to methods that can lead to participants exercising within the same exercise intensity domain, each of which are characterised by distinct V̇O_2_ kinetic and blood lactate profiles [19, 20]. This study utilised LT_1_ to identify the transition between moderate and heavy intensity domains, in accordance with the literature [19, 20]. However, a limitation within the current study was the use of LT_2_ to identify the heavy-severe domain transition, due to a lack of evidence supporting this, as well as the number of different methods used to measure and identify LT_2_ [7, 21]. As such, firm conclusions cannot be made regarding the heavy-severe domain transition from this study. It would have been more appropriate to measure CP/CS for this purpose [19–21], however, only data from a GXT were available in this study.

This highlights an important limitation to the widespread implementation of intensity domain-related exercise prescription. Specifically, the suitability of different testing protocols for identifying different threshold concepts, as well as data collection and threshold identification methods used [21]. This poses the challenge of how to simply prescribe domain-specific exercise intensity. Data from the present study may support the use of RPE for this purpose, as no difference in RPE was found between groups at LT_1_ (Fig. 1g) and LT_2_ (Fig. 1h), in support of previous findings [15]. Mean RPE at LT_1_ and LT_2_ in this study were found to be 11 and 15, respectively, suggesting that these values could be used to guide exercise prescription using a simple and easy to implement method. However, the SD for these values ranged from 1 to 2 units dependent on group. This inter-individual variation serves, therefore, as a limitation to the use of fixed RPE values for exercise intensity prescription and highlights the importance of individualisation in this context. Individualisation, though, poses a further challenge, due to the trade-off between the precision required for research purposes versus the simple messaging for population-level exercise guidelines.

### Implications for research

Our results highlight the need to prescribe exercise intensity in a way that ensures a homogenous intensity domain distribution between participants within both acute and longitudinal study designs. Previously, studies have conducted training interventions with an intensity of either a target range, or fixed value, using variables such as %V̇O_2peak_, %HR_peak_ and RPE, e.g. [22–26]., which will have led to significant domain heterogeneity. This means the dose of exercise stimulus would not have been controlled between participants. Moving forwards, researchers should identify the domain transitions and prescribe intensity in relation to these. That being said, the authors are not aware of any investigations into CP/CS using participants with SCI, so initial studies need to investigate the validity and reliability of identifying domain transitions in participants in SCI. Furthermore, V̇O_2_ kinetic responses to exercise in each domain should be investigated in participants with SCI, due to potential differences in V̇O_2_ kinetics between non-disabled participants and those with SCI [27]. Finally, these investigations must also account for any differences based on the mode of exercise [28].

Our findings also emphasise the need to individualise the exercise intensity prescription. In non-disabled participants, standardised intensity prescription (e.g., 55–75% V̇O_2peak_), has been shown to lead to significant heterogeneity in responsiveness [29] leading to participants being described as either “responders” or “non-responders” to the intervention. However, an individualised exercise prescription (relative to ventilatory threshold) in non-disabled participants resulted in 100% responsiveness, compared to 60% in the standardised intervention [30]. Subsequently, future exercise research in participants with SCI should individualise the intensity prescription according to intensity domains, whilst also report individual responsiveness to an intervention. This will improve methodological control while also increasing confidence in conclusions made based on the data.

### Implications for exercise guidelines using “moderate to vigorous” exercise intensity

Exercise guidelines must balance the precision required for a specific intensity prescription, against the need for a simple population-level recommendation. Furthermore, scientific guidelines must undergo a knowledge translation process to ensure that the scientific integrity of the guidelines are maintained, whilst also incorporating the varied needs of all potential end-users [31].

In adults with SCI, the scientific guidelines [1, 3] recommend performing aerobic exercise at a “moderate to vigorous” intensity, without providing any specific details on what this means. The same intensity prescription is also used in a community and clinical-practice version of the guidelines [31]. Results from the current study would suggest the scientific integrity of such an intensity prescription is questionable. Table 3 shows equivalent thresholds for PARA and TETRA for “moderate” and “vigorous” intensity, based on the guidelines for non-disabled adults [5]. Combining these with the intensity domain distributions in Fig. 2, shows participants were spread across moderate, heavy, and severe domains at both “moderate” and “vigorous” intensities. It should also be noted that participants in the present study were competitive athletes, and that responses would likely be even more variable for sedentary or low-active populations. This shows how the use of “moderate” to “vigorous” intensity will not lead to anything close to resembling a uniform exercise intensity prescription between individuals. It also shows how someone expecting to perform “moderate” intensity exercise may actually be much closer to their maximum capacity. This will likely decrease the pleasure the person feels during the exercise, which could ultimately impact on whether they decide to continue doing it [32].

As it is operationally more difficult to define compared to frequency (e.g., 3 times a week) and duration (e.g., 30 min), it is possible that exercise intensity becomes an ignored piece of exercise guidelines. Perhaps research could seek to understand end-user perceptions of “exercise intensity”, or needs for interpreting and monitoring intensity, before using that information alongside physiological principles to underpin an evidence-informed intensity prescription. Alternatively, maybe the focus for exercise guidelines should shift from exercise intensity to also acknowledge factors that might help individuals become and stay active, such as their pleasure when performing exercise [32].

## Conclusion

Prescribing a “moderate to vigorous” exercise intensity will not lead to a uniform intensity domain distribution in adults with SCI. Neither will the use of fixed percentages of V̇O_2peak_ or HR_peak_, or generic values of RPE, due to inter-individual variation. Such methods of exercise intensity prescription should not be used in this population. Future research should individualise the intensity prescription to ensure a homogenous inter-individual domain distribution. However, the accurate testing required to ensure an individualised intensity prescription poses a challenge to exercise guidelines aimed at informing behaviour at the population-level.

### Data archiving

The datasets generated and analysed during the current study are available from the corresponding author on reasonable request.

## Supplementary information

Supplementary material
