# Supplementary material for: Rethinking aerobic exercise intensity prescription in adults with spinal cord injury: time to end the use of “moderate to vigorous” intensity?
Source: Spinal Cord. 2021 Dec 8;60(6):484–90. doi: 10.1038/s41393-021-00733-2 (PMC9209328; doi:10.1038/s41393-021-00733-2)
Supplement: Supplementary file 1 — Supplementary material [file 41393_2021_733_MOESM1_ESM.pdf]

Hutchinson MJ, Goosey-Tolfrey VL, Rethinking aerobic exercise intensity prescription in adults with spinal cord injury: time to end the use of "moderate to vigorous" intensity?

Supplementary Material 1: Dynamic model with lagged independent variable for RPE and %$\dot{V}O_{2peak}$

$x = \% \; \dot{V}O_{2peak}$

$y =$ RPE

Z1 = 1, when i = 1 (i = measurement occasion)

Z2 = 1, when i > 1

TETRA = 1, if Group = TETRA

$$y_{ij} = \beta_{1j}Z1_{ij} + \beta_{2j}Z1.x_{ij} + \beta_{3i}Z2_{ij} + \beta_{4j}Z2.x_{ij} + \beta_{5j}Z2.x_{i-1j} + \beta_{6i}Z2.TETRA_j + u_{0j}$$

$$\beta_{1j} = \beta_1 + e_{1ij}$$

$$\beta_{2j} = \beta_2 + u_{2j}$$

$$\beta_{3i} = \beta_3 + e_{3ij}$$

$$\beta_{4j} = \beta_4 + u_{4j}$$

$$\beta_{5j} = \beta_5 + u_{5j}$$

$$\beta_{6i} = \beta_6 + e_{6ij}$$

$$\begin{pmatrix} u_{0j} \\ u_{2j} \\ u_{4j} \\ u_{5j} \end{pmatrix} \sim N(0, \Omega_u) : \Omega_u = \begin{bmatrix} \sigma^2_{u0} & & & \\ \sigma_{u02} & \sigma^2_{u2} & & \\ \sigma_{u04} & 0 & \sigma^2_{u4} & \\ \sigma_{u05} & 0 & \sigma_{u45} & \sigma^2_{u5} \end{bmatrix}$$

$$\begin{pmatrix} e_{1ij} \\ e_{3ij} \\ e_{6ij} \end{pmatrix} \sim N(0, \Omega_e) : \Omega_e = \begin{bmatrix} \sigma^2_{e1} & & \\ 0 & \sigma^2_{e3} & \\ 0 & \sigma_{e36} & \sigma^2_{e6} \end{bmatrix}$$

$$y_{ij} = 5.133Z1_{ij} + 0.074Z1.x_{ij} + 3.411Z2_{ij} + 0.093Z2.x_{ij} + 0.074Z2.x_{i-1j} - 1.081Z2.TETRA_j + u_{0j}$$

$$-2loglikelihood = 2744.893$$

$$\begin{pmatrix} u_{0j} \\ u_{2j} \\ u_{4j} \\ u_{5j} \end{pmatrix} \sim N(0, \Omega_u) : \Omega_u = \begin{bmatrix} 3.524\ (0.459) & & & \\ -0.047\ (0.009) & 0.001\ (0.000) & & \\ 0.000 & 0 & 0.000 & \\ 0.000 & 0 & 0.000 & 0.000 \end{bmatrix}$$

$$\begin{pmatrix} e_{1ij} \\ e_{3ij} \\ e_{6ij} \end{pmatrix} \sim N(0, \Omega_e) : \Omega_e = \begin{bmatrix} 0.768\ (0.381) & & \\ 0 & 1.044\ (0.069) & \\ 0 & 0.212\ \ (0.126) & 0.000 \end{bmatrix}$$

Level 1 variance:

$$Z1 = \sigma_{e1}^2 = 0.768$$

$$Z2\ (PARA) = \sigma_{e3}^2 = 1.044$$

$$Z3 = \sigma_{e3}^2 + 2\sigma_{36} + \sigma_{e6}^2 = 1.469$$

Level 2 variance

$$Z1 = \sigma_{u0}^2 + 2\sigma_{u02} + \sigma_{u2}^2 = 3.431$$

$$Z2 = \sigma_{u0}^2 + 2\sigma_{u04} + \sigma_{u4}^2 + 2\sigma_{u05} + 2\sigma_{u45} + \sigma_{u5}^2 = 3.524$$

Coefficient	Value	Standard error	P
β_1	5.113	0.473	< 0.0005
β_2	0.074	0.013	< 0.0005
β_3	3.411	0.242	< 0.0005
β_4	0.093	0.009	< 0.0005
β_5	0.074	0.010	< 0.0005
β_6	-1.081	0.419	0.009

Hutchinson MJ, Goosey-Tolfrey VL, Rethinking aerobic exercise intensity prescription in adults with spinal cord injury: time to end the use of "moderate to vigorous" intensity?

Supplementary Material 2: Dynamic model with lagged independent variable for RPE and %HR$_{peak}$

x = % HR$_{peak}$

y = RPE

Z1 = 1, when i = 1 (i = measurement occasion)

Z2 = 1, when i > 1

PARA = 1, if Group = PARA

$$y_{ij} = \beta_{1j}Z1_{ij} + \beta_2 Z1.x_{ij} + \beta_{3i}Z2_{ij} + \beta_4 Z2.x_{ij} + \beta_5 Z2.x_{i-1j} + \beta_{6i}PARA + u_{0j}$$

$$\beta_{1j} = \beta_1 + e_{1ij}$$

$$\beta_{3i} = \beta_3 + e_{3ij}$$

$$(u_{0j}) \sim N(0, \Omega_u): \Omega_u = [\sigma_{u0}^2]$$

$$\begin{pmatrix} e_{1ij} \\ e_{3ij} \end{pmatrix} \sim N(0, \Omega_e): \Omega_e = \begin{bmatrix} \sigma_{e1}^2 & \\ 0 & \sigma_{e3}^2 \end{bmatrix}$$

$$y_{ij} = -1.375Z1_{ij} + 0.160Z1.x_{ij} - 3.044Z2_{ij} + 0.168Z2.x_{ij} + 0.044Z2.x_{i-1j} + 0.707PARA + u_{0j}$$

$$-2loglikelihood = 2727.763$$

$$(u_{0j}) \sim N(0, \Omega_u): \Omega_u = [2.929\ (0.387)]$$

$$\begin{pmatrix} e_{1ij} \\ e_{3ij} \end{pmatrix} \sim N(0, \Omega_e): \Omega_e = \begin{bmatrix} 1.471\ (0.209) & \\ 0 & 1.182\ (0.073) \end{bmatrix}$$

Coefficient	Value	Standard error	P
β_1	-1.375	0.814	0.091
β_2	0.160	0.014	< 0.0005
β_3	-3.044	0.355	< 0.0005
β_4	0.168	0.010	< 0.0005
β_5	0.044	0.011	< 0.0005
β_6	0.707	0.324	0.029
